# Surveillance of *Clostridium difficile* Infections: Results from a Six-Year Retrospective Study in Nine Hospitals of a North Italian Local Health Authority

**DOI:** 10.3390/ijerph14010061

**Published:** 2017-01-10

**Authors:** Greta Roncarati, Laura Dallolio, Erica Leoni, Manuela Panico, Angela Zanni, Patrizia Farruggia

**Affiliations:** 1 Unit of Microbiology, Sant’Orsola-Malpighi Hospital, Via Massarenti 9, Bologna 40138, Italy; greta.roncarati@ausl.bologna.it; 2 Unit of Hygiene, Public Health and Medical Statistics, Department of Biomedical and Neuromotor Sciences, University of Bologna, Via San Giacomo 12, Bologna 40126, Italy; laura.dallolio@unibo.it; 3 Direction of Maggiore Hospital, Local Health Authority of Bologna, Via Largo Nigrisoli 2, Bologna 40133, Italy; m.panico@ausl.bologna.it; 4 Unit of Hygiene and Quality of Residential Services, Bellaria Hospital, Local Health Authority of Bologna, Via Altura 3, Bologna 40139, Italy; angela.zanni@ausl.bologna.it (A.Z.); patrizia.farruggia@ausl.bologna.it (P.F.)

**Keywords:** *Clostridium difficile*, healthcare associated infections, hospital surveillance

## Abstract

*Clostridium difficile* is an emerging cause of healthcare associated infections. In nine hospitals of an Italian Local Health Authority the episodes of *C. difficile* infection (CDI) were identified using the data registered by the centralized Laboratory Information System, from 2010 to 2015. CDI incidence (positive patients for A and/or B toxins per patients-days) was analysed per year, hospital, and ward. A number of cases approximately equivalent to the mean of identified cases per year were studied retrospectively to highlight the risk factors associated to CDI and their severity. Nine hundred and forty-two patients affected by CDI were identified. The overall incidence was 3.7/10,000 patients-days, with a stable trend across the six years and the highest rates observed in smaller and outlying hospitals (up to 17.8/10,000), where the admitted patients were older and the wards with the highest incidences (long-term-care: 7.6/10,000, general medicine: 5.7/10,000) were more represented. The mean age of patients in each hospital was correlated with CDI rates. Of the 101 cases selected for the retrospective study, 86.1% were healthcare associated, 10.9% community acquired; 9.1% met the criteria for recurrent case and 23.8% for severe case of CDI. The overall mortality rate was 28.7%. Comorbidity conditions occurred in 91.1%, previous exposure to antibiotics in 76.2%, and proton pump inhibitors in 77.2%. Recurrent and severe cases were significantly associated with renal insufficiency and creatinine levels ≥2 mg/dL. The survey based on the centralized laboratory data was useful to study CDI epidemiology in the different centres in order to identify possible weaknesses and plan control strategies, in particular the reinforcement of staff training, mainly targeted at compliance with contact precautions and hand hygiene.

## 1. Introduction

Over the last two decades *Clostridium difficile* has been reported as the greatest cause of healthcare associated infections in North America and Europe [1,2,3,4].

Clinical manifestations range from mild diarrhoea to pseudomembranous colitis, toxic megacolon, bowel perforation, and death [5,6]. The increasing morbidity and mortality of *C. difficile* infections (CDIs) are attributable to emerging highly virulent strains, known as ribotype 027 (commonly referred to as 027/NAP1/B1) and ribotype 078, which have been implicated in severe hospital outbreaks in the United States (U.S.), Canada, and Europe [7,8,9,10,11].

Major risk factors of CDI include hospitalization and prolonged length of stay in hospital, advanced age (>65 years), and antibiotic exposure (in particular to clindamycin, fluoroquinolones, and cephalosporins) within the preceding three to four months [6,12,13,14]. Other secondary risk factors include comorbidity conditions such as renal disorders [15,16], inflammatory bowel diseases [17], acid reducing therapy [2,18,19], and gastrointestinal surgery or procedures [12].

In U.S. hospitals, from 2001 to 2010, the overall CDI incidence was 5.9 per 1000 total discharges, with a mortality rate of 8.8% in older adults, 6.9% in adults, and 3.1% in the paediatric population [20]. CDI incidence increased from 5.6 per 1000 discharges in 2001 to 12.7 per 1000 discharges in 2011 [21]. In Europe, a study conducted on 482 hospitals across 20 European countries in 2012–2013 reported 7.0 cases of CDI per 10,000 patients-days (country range from 0.7 to 28.7) [22]. The overall incidence rates showed an increasing trend compared with 2005 (2.5 cases per 10,000 patients-days) [23] and 2008 (4.1 per 10,000 patients-days) [24]. However, a high variability among European hospitals and countries was observed, with increasing incidence rates in Spain [25], Germany [26], and France [27], while the number of cases remained static in Belgium and decreased in England and Wales from 2007–2008 [3]. This reduction may be attributable to the implementation of prevention strategies and the introduction of mandatory reporting of CDI after a six-fold increase of CDI related mortality had been observed from 1999–2006 [28].

In Italy, a mandatory surveillance system for CDI is not active and it is difficult to have an overall picture of CDI incidence. Italian data regarding CDI rates derive mainly from some European surveys conducted in 2005 [23], 2008 [24], and 2012–2013 [22], involving few Italian hospitals. Other studies report local data from hospitals in North and Central Italy: the incidence rates range from less than 1 to 23.3 per 10,000 patients-days [29,30,31,32,33]; however, these data are not comparable, since they include different wards and categories of patients and are derived from a wide variety of diagnostic procedures and surveillance methods across hospitals [34]. The overall analysis of the available data shows that the incidence of CDI significantly increased from 2006 to 2014 and that the general medicine wards have the highest number of cases, with percentages ranging from 46% to 80% of cases [29,30,31], and incidence rates up to 23.3 per 10,000 patients-days [32]. Ribotype 027 is also emerging in Italy and a cluster of fulminant *C. difficile* 027 colitis was recently observed in an intensive care unit in Rome [35].

Following the increase in the incidence and the emergence of the 027 strain, different guidelines for the diagnosis, treatment, and prevention of CDI were published [36,37,38]. In Italy the SIMPIOS (Società Italiana Multidisciplinare Prevenzione Infezioni Organizzazioni Sanitarie) prepared a guideline document which, on the basis of the literature and international guidelines, suggests prevention strategies and the adoption of an active surveillance system in hospitals, making it possible to quantify the impact of these infections and assess the success/failure of the measures adopted [39].

In the nine hospitals included in its territory, the Local Health Authority of Bologna (Emilia Romagna Region) implemented a system of surveillance and control of a number of pathogens, identified as “alert organisms”, such as *C. difficile*. The aim of this study was to assess the CDI incidence in these healthcare facilities from 1 January 2010 to 31 December 2015. A number of cases approximately equivalent to the mean of identified cases per year was selected in order to study retrospectively the risk factors associated with CDI onset and their severity.

## 2. Materials and Methods

### 2.1. Setting and Study Design

The study was conducted in the nine hospitals of the Local Health Authority of Bologna, Emilia Romagna Region, Italy. The territory of the Local Health Authority extends approximately over 3000 km^2^ and includes about 868,000 inhabitants, 23% over 65 and 8% over 80 years. Of the nine hospitals, two are in the city centre and the remaining seven are in the surrounding areas, accounting for a total of around 230,000 admissions per year. From 1 January 2010 to 31 December 2015 all patients with CDI were identified through the hospital laboratory database.

The study was performed within the institutional surveillance of healthcare-associated infections and involved the analysis of existing anonymised clinical and laboratory data. An informed consent for the use of anonymised data for scientific purposes was signed by all patients admitted to the hospitals and for this reason an ethical approval was not required.

The incidence of CDI was calculated for the six years of the study, stratified according to type of ward and hospital. A retrospective study was carried out on a number of cases approximately equivalent to the mean of identified cases per year in the wards of general medicine, long-term care, intensive care unit (ICU), surgery, and other wards (cardiology, neurology, etc.) using the patients’ clinical charts to collect data on epidemiological, clinical, and microbiological characteristics in order to assess the risk factors present and the causes of the severe and recurrent cases.

### 2.2. Definitions

According to the recommended Italian guidelines [39], a case of CDI was defined as the presence of liquid or informed stools or toxic megacolon and laboratory analysis positive for *C. difficile* toxin A and/or B. All other causes of diarrhoea were excluded as well as asymptomatic patients with *C. difficile*, even if positive for toxin. CDI was assumed to be healthcare associated if diarrhoea started from 48 h after hospital admission to four weeks after hospital discharge or within 48 h from admission of patients discharged from another healthcare facility within the previous four weeks. Cases were defined as community-acquired if CDI signs occurred within 48 h from admission of patients who had not stayed in another hospital/healthcare facility in the previous three months. Cases that did not fit any of these criteria were classified as unknown [39].

A case was defined as recurrent if the symptoms, accompanied by a *C. difficile* positive test, recurred no more than eight weeks after the complete resolution of the first episode. A case was defined as severe when at least one of the following events occurred within 30 days of onset of symptoms: admission to ICU for complications, colectomy for toxic megacolon, perforation, and death [39].

### 2.3. Data Collection

In the hospitals of the Local Health Authority of Bologna, infection surveillance is supported by a system (MERCURIO, Noemalife, Bologna, Italy) which provides reliable information in a timely manner to effectively identify the infective strains. This system acquires essential microbiology data directly gleaned from the Laboratory Information System (LIS) and patient information such as admission data and clinical information.

For the computation of the annual incidences of CDI, cases identified through LIS were related to the overall number of hospital patients-days obtained from the hospital archives. If at least two patients with CDI were identified in the same operative unit, an epidemiologic investigation was initiated involving the collection of anamnestic and clinical-assistential data in order to check for a possible association between the cases and thus the presence of epidemic clusters.

For the retrospective analysis of the risk factors for CDI, recurrent CDI, and severe CDI, data were collected from the patients’ clinical charts. From the 942 cases that occurred during the six years of the study, 148 were randomly selected, approximately equivalent to the mean incidence per year in the selected wards (general medicine, long-term care, intensive care unit, surgery, cardiology, neurology). Patients were selected from all nine hospitals. However, these were not all aligned with the digital clinical charts system: the smaller outlying hospitals had only gradually adopted the computerized archive system, whereas it had been used from the beginning of the study in the larger urban hospitals. Of the 148 selected cases, only 101 clinical charts were available, corresponding to approximately 10% of the total cases. The loss of medical records was concentrated on small outlying hospitals, especially in the early years of the study.

A database was set up for the collection of all the items related to: epidemiological characteristics of patients (age, gender), clinical history (comorbidities, gastrointestinal surgery, previous hospitalization within 30 days before CDI, previous therapy with antimicrobials, proton pump inhibitors, and/or histamine 2 blockers within 30 days before CDI, length of hospital stay before developing CDI), symptoms and therapeutic management (duration of diarrhoea, serum albumin ≤ 2.5 g/dL, serum creatinine ≥ 2 mg/dL, and/or leukocyte count ≥ 20 × 10^9^/L during four days before or two days after CDI, type of antimicrobial treatment and complications). Moreover, the number of deaths was recorded, reporting the cause of death and the contribution of CDI to each death, as reported by the clinicians on the clinical charts.

### 2.4. Microbiological Analysis

All the microbiological analyses were performed in the same centralized laboratory of the Local Health Authority. Screening for *C. difficile* was performed using a rapid enzyme immunoassay: the Tox A/B Quik Chek^®^ test (Wampole™), which searches for toxins A and B in faecal specimens from persons suspected of having *C. difficile* disease, according to clinical manifestation. Positive samples were treated by ethanol shock, inoculated onto a selective medium (Clostridium Difficile Agar Base, supplemented with taurocholate 1 g/L, cycloserine 500 mg/L and cefoxitin 16 mg/L, Oxoid, Basingstoke, UK), and incubated anaerobically at 37 °C for 48 h. *C. difficile* was identified by characteristic colony morphology and Gram stain. The positive cultures were confirmed with a rapid latex agglutination test (*C. difficile* Test Kit, Oxoid, Basingstoke, UK).

### 2.5. Statistical Analysis

Incidence rates were calculated as the number of patients with positive *C. difficile* toxin assay per 10,000 patients-days. In order to compare annual rates, 95% confidence intervals (CIs) were calculated, assuming a Poisson distribution [40]. For trend analysis we used χ^2^ test for trend. Categorical variables were compared among severe CDI cases, recurrent CDI cases, and non-severe and non-recurrent CDI cases using Pearson χ^2^ test or Fisher’s exact test, while continuous independent variables were compared among these groups using Kruskal-Wallis test.

The significance level was set to *p* < 0.05. Multinomial logistic regression was used to analyse the risk factors of severe CDI and recurrent CDI. In this analysis non-severe and non-recurrent CDI cases were used as reference group.

All statistical analyses were conducted using the SPSS Statistics, Version 22 (IBM, Chicago, IL, USA) for Windows, and Stata, using the *p*-test procedure to calculate χ² for trend.

## 3. Results

### 3.1. CDI Rates

From January 2010 to December 2015, a total of 942 CDI cases were identified with an overall incidence of 3.7 per 10,000 patients-days without significant trends in the annual incidence (Table 1). The number of samples tested per 10,000 patients-days did not vary significantly during the study period and was on average 27.1/10,000. The percentage of positive tests was stable across the years ranging from 12% in 2013 (the lowest value) to 14.9% in 2010 (the highest value during the study period). The positive result of the toxin test was confirmed by bacterial culture in over 96% of cases.

The incidence of CDI cases showed a high variability among the nine hospitals (named from H1 to H9 in decreasing order of size), ranging from the highest values in H9 and H5 (overall incidence across years: 17.8/10,000 and 8.6/10,000, respectively) and the lowest values in H4 (1.8/10,000), H1 (2.4/10,000), and H2 (2.7/10,000). The hospitals with the highest incidences (H9 and H5) were also those that requested the greatest number of laboratory tests, and had the highest percentages of positive tests (around 20% vs. 11%–14% in the other hospitals) (Table 2).

**Figure 1:**
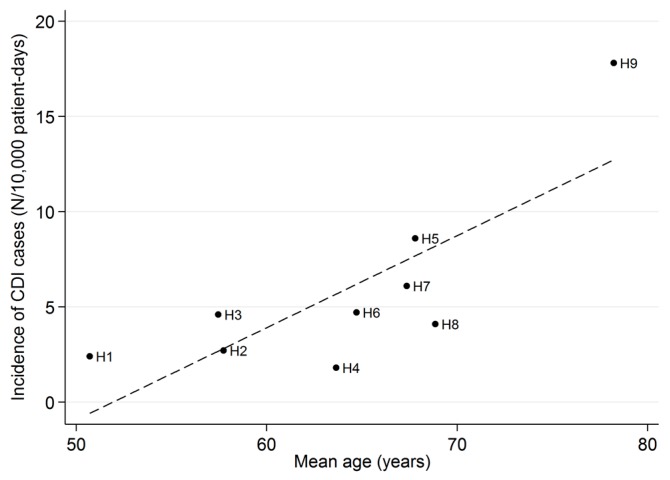
Overall incidences of CDI in the nine hospitals in relation to the mean age of the hospitalized patients.

Hospital H9, which had by far the highest incidence, is a hospital in the mountainous zone with wards only in the area of medicine (long-term care and general medicine). In the outlying hospitals the mean age of hospitalized patients, based on the hospital discharge records, was greater than those of the two urban hospitals. The overall incidence of CDI in the various hospitals correlated positively with the mean age of the patients (r = 0.78; *p* < 0.01) (Figure 1).

**Figure 2:**
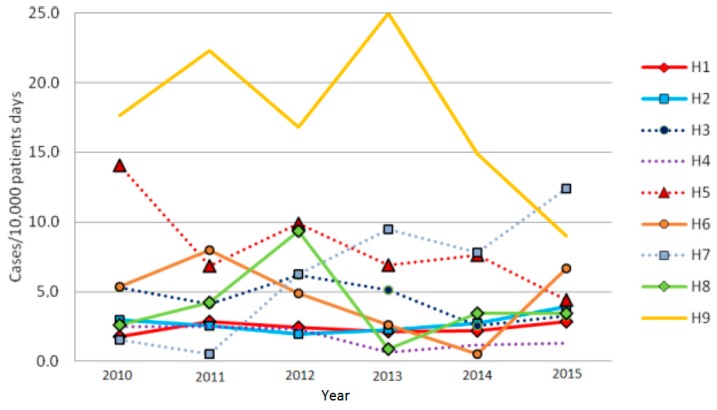
Trends of CDI incidences per year in the nine hospitals of the Local Health Authority of Bologna.

Figure 2 shows the trend of the incidences per year in the nine hospitals. In the whole period 20 outbreaks were observed, with nine episodes in 2010 and two to three events per year in the remaining years (Table 2). The two hospitals in the urban area (H1 and H2) were larger, and therefore showed a regular trend of incidences over time, little influenced by the occurrence of outbreaks. Instead, in the smaller hospitals some peaks can be observed in coincidence with years when some epidemic clusters occurred. The two peaks observed in 2011 and 2013 in hospital H9 can be accounted for by two clusters that occurred in the long-term care wards, both with 10 cases, and similarly the peak seen in hospital H5 in 2010 coincided with a cluster of nine cases that again occurred in the wards of long-term care (Figure 2).

Table 3 shows the overall distribution of CDI cases during the study period, stratified per ward, in the nine hospitals of the Local Health Authority of Bologna. Around 88% of cases occurred in the area of medicine (general medicine, long-term care wards) with the highest number of cases in general medicine (53.4%) and the highest incidence rate in long-term care (7.6/10,000).

### 3.2. Patients’ Characteristics

Table 4 shows the epidemiological and clinical characteristics of the CDI cases selected for the retrospective study on risk factors. Almost all patients were over 65 years of age, with a very high median age (82 years); about two thirds were women. Most cases were healthcare associated (86.1%), while only 10.9% were community acquired, leaving 3% of indeterminate association.

The median of length of stay was 28 days. For patients with confirmed healthcare associated CDI, the median interval between admission and microbiological diagnosis was 15 days, with an interquartile range between 7 and 29 days. CDI was more frequently diagnosed in general medicine and long-term care wards (respectively 49.5% and 35.6%).

For each patient a comorbidity index was assigned, equal to the number of comorbidities present. The global comorbidity index is the mean of comorbidities present in all CDI cases. A large proportion of patients had comorbidity conditions (91.1%) and the global comorbidity index was 2.0 (SD: 1.2). A previous exposure to antibiotics or proton pump inhibitors was observed in 76.2% and 77.2% of cases, respectively. In one third of patients the duration of diarrhoea was longer than one week and in nine of these cases diarrheal stools were mixed with blood. Elevated peripheral leukocyte count, high serum creatinine, and low serum albumin occurred in about one fifth of patients. A total of 84 patients (83.2%) were specifically treated for CDI, mainly with metronidazole.

Ten patients met the criteria for recurrent case (9.1%) and twenty-four for severe case (23.8%). Death occurred in 29 patients (28.7%); in two of these cases CDI was considered the primary cause (6.9%) and in 27 patients CDI was a contributing cause of death (93.1%).

### 3.3. Risk Factors for Recurrent and Severe Cases

Table 5 shows the results of the bivariate analyses of risk factors for recurrent and severe CDI cases. The statistically significant factors (*p* < 0.01) were chronic renal insufficiency and the detection of a level of serum creatinine higher than 2 mg/dL within four days before or two days after the CDI episode.

Table 6 shows the results of multinomial logistic regression analyses of risk factors for severe CDI and recurrent CDI. As in the bivariate analysis, the detection of a serum creatinine higher than 2 mg/dL remained significantly associated both with severe and recurrent CDI (respectively OR 7.15, 95% CI: 2.03–25.16 and OR 5.33, 95% CI: 1.02–27.93), while chronic renal insufficiency was significantly associated only for recurrent CDI cases (OR 6.20, 95% CI: 1.09–35.41).

## 4. Discussion

Healthcare associated infections are an emerging problem for public health. The increase in the number of cases and outbreaks related to drug resistant bacteria [39,41,42] led the Committee for Hospital Infection of the Local Health Authority of Bologna to implement surveillance systems. Since 2010 a surveillance system for *C. difficile* infections has been implemented, based on the Laboratory Information System.

From 2010 to 2015 a stable trend in the annual incidence of CDI was observed: overall 3.7/10,000 patients-days. This is consistent with the data reported for the Emilia Romagna Region (4/10,000 patients-days) [43] and in other Italian hospitals [29,30,31]. Also, the average percentage of positive laboratory test for toxins (13.6%) was close to that of the 12.2% estimated at national level [34]. However, a high variability in CDI rates was observed between the different hospitals. The differences observed can be explained by local conditions. The large urban hospitals had different spectra of admission diagnoses compared to the smaller ones. In the outlying hospitals the wards were less specialized, the higher risk wards (general medicine and long-term care) were more represented, and the patients were older. In addition, it is possible that compliance with the standards of care differed among the staff of the various health care facilities. Moreover, the incidence of CDI in the smaller hospitals varied considerably over the years due to the occurrence of clusters which, on account of the lower denominators (patients-days), had a marked influence on the annual rates. On the other hand, in the two largest hospitals, situated in the urban area (H1 and H2), the CDI rates were stable over time (2.4–2.7/10,000, respectively), and were lower than the overall mean value. The area of medicine (general medicine and long-term care wards) was the most affected by episodes of CDI, comprising around 88% of cases, followed by the surgery (4.1%) and the ICU (1.8%), in accordance with the data reported in other Italian studies [29,31,33,43].

The retrospective study on epidemiological and clinical characteristics of the patients confirmed the high prevalence of the known risk factors for CDI, such as older age, the use of antimicrobials and proton pump inhibitors, and hospital stay before CDI [2,6,13,32], while levels of serum creatinine higher than 2 mg/dL and chronic renal insufficiency were predictive factors for severe and recurrent CDI cases, similar to what was found by other authors [15,16]. Although the occurrence of some known and well-recognized risk factors (i.e., leucocytosis and hypoalbuminemia) was higher in severe cases compared with non-severe cases (29.2% vs. 17.9% and 44.4% vs. 22.6%, respectively), these conditions were not found to be statistically associated with severe disease, probably due to the low number of cases included in the retrospective study. The overall mortality rate was 28.7%, higher than the rate reported by most studies [13,27,32], but similar to the findings of Alicino et al. [29] in CDI cases with a very high median age (81 years), comparable to that of our cases (82 years).

Some weaknesses of this study should be considered. First, the incidence of CDI was possibly underestimated since the enzyme immunoassay test used for the detection of toxins (Tox A/B Quik Chek^®^ test-Wampole^™^) has a high specificity, around 95%, also confirmed in our study, but a low sensitivity, around 55% [44]. Another limitation is due to the low number of participants to the retrospective study. Almost one third of all patients initially selected were lost, due to the unavailability of clinical charts, especially in outlying hospitals, where the computerized archive system was not yet active in the first period of the study. Consequently, 10% of patients included in the study were not equally representative of all hospitals. The retrospective study was limited to a description of the distribution of the known risk factors and analysed the predictive value of the risk conditions for severe and recurrent cases in a population of patients who were very elderly, made great use of antibiotics, and had many comorbidities.

Despite these limitations, in the absence of mandatory reporting of CDI in Italy, this laboratory-based surveillance system proved to be useful in monitoring the trend in the annual incidence and clusters in the different healthcare facilities, as well as the number of tests performed and the percentage of positive tests. In particular, the percentages of test positivity attained in the various hospitals showed no statistically significant differences. This can be seen as evidence of similar attitudes among medical staff in considering the inclusion of patients and requesting a stool specimen. When considering different hospitals with different medical staff, it is important to ascertain that medical and nursing personnel adopt the same protocol, in order to make the results comparable.

The LIS based surveillance system was also useful for identifying areas where a corrective intervention was needed, for example, modifying the protocol of patient handling, adopting extraordinary measures of environmental disinfection, or implementing staff training. Consequent to the survey, visits and inspections were implemented in the wards with high rates of CDI and/or epidemic clusters in order to verify compliance with the best practices and, where deficient, to allow reinforcement of staff training, mainly targeted at compliance with contact precautions, and the methods used for hand hygiene and for cleaning/disinfection of the surfaces. The differences observed in CDI rates between the different hospitals can be explained by local conditions, in particular, by the different distribution of the wards (medicine and long-term care are more represented in the smaller and outlying hospitals) and the characteristics of the patients, especially the mean age, which was positively correlated to the CDI rates.

## 5. Conclusions

As the laboratory methods can have a significant impact on CDI incidence [27,34], the use of a single laboratory for microbiological analysis implementing a unique protocol improves the monitoring of CDI episodes and helps in understanding their epidemiological trend. Furthermore, it is important that clinicians should be instructed to adopt the same criteria for requesting laboratory diagnosis in the event of clinical suspicion.

In this study, the laboratory-based surveillance system was useful in monitoring the trend of CDI in the nine hospitals of the Local Health Authority of Bologna. The differences observed between the various hospitals were mainly due to the different distribution of the wards (general medicine and long-term care were more represented in the smaller and outlying hospitals) and the characteristics of the patients, especially the mean age, which was positively correlated with the CDI rates. The finding that a history of chronic renal failure or an elevated serum creatinine were predictors of severe and/or recurrent disease is consistent with the data reported by other studies [15,16]. Despite its limited external validity, the surveillance system adopted presents internal validity and has proved to be a useful tool to identify the weaknesses of the various hospitals and wards, to define the areas of intervention, and to plan the necessary control strategies.

## Figures and Tables

**Table 1:** Stool samples tested (numbers and incidence rates) and *Clostridium difficile* infection (CDI) cases (numbers and incidence rates) per 10,000 patients-days, per year.

Years	Tested Stool Samples				CDI Cases				
	*n*	*n*/10,000 Patients-Days	95% CI	*p* Trend	% Positive of Taken Samples	*n*	*n*/10,000 Patients-Days	95% CI	*p* Trend
2010	1172	25.3	23.8–26.7	ns	14.9	175	3.8	3.2–4.4	ns
2011	1279	27.0	25.5–28.5		14.2	181	3.8	3.3–4.4	
2012	1366	29.8	28.3–31.4		13.5	185	4.0	3.5–4.7	
2013	1182	29.3	27.7–31.1		12	142	3.5	3.0–4.2	
2014	922	23.8	22.3–25.4		12.6	116	3.0	2.5–3.6	
2015	1007	26.9	25.3–28.6		14.2	143	3.8	3.2–4.5	

CI: confidence interval; ns: not significant.

**Table 2:** Overall incidence of CDI in the nine hospitals of the Local Health Authority of Bologna during the study period (2010–2015). Hospitals are numbered in decreasing size (patients-days).

Hospitals	Tested Stool Samples		CDI Cases			Outbreaks	
	*n*/10,000 Patients-Days	% Positive of Taken Samples	*n*	*n*/10,000 Patients-Days	95% CI	*n*	Number of Cases per Outbreak
H1	22.5	10.8	264	2.4	2.1–2.7	4	5–9
H2	24.3	11.4	125	2.7	2.2–3.1	1	2
H3	31.1	14.0	127	4.6	3.8–5.4	3	5–12
H4	14.8	12.7	36	1.8	1.3–2.5	1	4
H5	40.1	20.8	130	8.6	7.2–10.3	2	6–9
H6	33.1	14.3	53	4.7	3.5–6.1	2	3–8
H7	38.9	14.1	67	6.1	4.7–7.7	2	5–12
H8	27.6	14.5	28	4.1	2.7–5.9	0	-
H9	83.3	22.0	112	17.8	14.7–21.4	5	3-10

**Table 3:** Overall incidence of CDI stratified per wards, 2010–2015.

Wards	CDI Cases				Outbreaks	
	*n*	%	*n*/10,000 Patients-Days	95% CI	*n*	Number of Cases per Outbreak
General Medicine	503	53.4	5.7	5.2–6.3	7	4–12
Long-Term Care	327	34.7	7.6	6.8–8.5	11	3–12
Surgery	39	4.1	0.7	0.4–0.9	0	-
Cardiology	23	2.4	2.0	1.3–3.0	1	2
Intensive Care Unit	17	1.8	2.3	1.4–3.8	1	8
Paediatrics	1	0.1	0.1	0.0–0.5	0	-
Others	32	3.4	1.1	0.8–1.6	0	-

CI: confidence interval.

**Table 4:** Epidemiological and clinical characteristics, therapeutic management, and outcomes of CDI patients (retrospective study).

Patients’ Features	Patients (*n* = 101)	
	*n*	%
**Epidemiological characteristics**		
Age, years, median (IQR)	82 (78–87)	
Age > 65 years	97	96.0
Gender, male	38	37.6
Epidemiological association		
Healthcare associated	87	86.1
Community acquired	11	10.9
Indeterminate association	3	3.0
Length of hospital stay, days, median (IQR)	28 (15–43)	
Length of hospital stay before CDI, days, median (IQR)	13 (5–26)	
Ward of admission		
General medicine	50	49.5
Long-term care	36	35.6
Intensive care unit	2	2.0
Surgery	4	4.0
Others	9	8.9
Comorbidity		
Chronic obstructive pulmonary disease	45	44.6
Heart failure	42	41.6
Chronic renal insufficiency	35	34.7
Diabetes	31	30.7
Malignant disease	12	11.9
Inflammatory bowel disease	8	7.9
Gastrointestinal surgery	14	13.9
Use of nasogastric tube feeding during the hospital stay	10	9.9
Previous hospitalization in the 30 days before CDI	35	34.7
Having at least one of the previous comorbidities	92	91.1
Medication exposures in the 30 days before CDI		
Any antibiotic not directed at CDI	77	76.2
Proton pump inhibitor	78	77.2
Histamine 2 blocker	13	12.9
**Clinical characteristics, therapeutic management, and outcomes**		
Leucocyte count ≥ 20 × 10^9^ cells/L *	20	19.8
Creatinine ≥ 2 mg/dL *	23	22.8
Albumin ≤ 2.5 mg/dL *	23	22.8
No therapeutic management	17	16.8
Metronidazole	56	66.7
Vancomycin	23	27.4
Other antibiotic	5	5.9
Recurrent CDI	10	9.1
Severe CDI	24	23.8
Intra-hospital all-cause mortality	29	28.7

IQR: interquartile range; * within four days before or two days after CDI.

**Table 5:** Bivariate analysis of risk factors for recurrent and severe CDI.

Patients’ Features	Non-Severe and Non-Recurrent CDI (*n* = 67)	Recurrent CDI (*n* = 10)	*p-*Value ^†^	Severe CDI (*n* = 24)		*p-*Value ^†^
**Epidemiological characteristics**						
Age, years, median (IQR)	81 (78–87)	81 (77–84)	ns	83 (78–88)		ns
Age > 65 years (%)	95.5	90.0	ns	100.0		ns
Gender, male (%)	38.8	20.0	ns	41.7		ns
Epidemiological association (%)						
Healthcare associated	86.5	70.0	ns	91.7		ns
Community acquired	11.9	10.0	ns	28.3		
Indeterminate association	1.5	20.0	ns	0.0		
Length of hospital stay, days, median (IQR)	30 (18–43)	26 (20–46)	ns	24 (11–41)		ns
Length of hospital stay before CDI, days, median (IQR)	14 (6–27)	4 (2–16)	ns	16 (6–30)		ns
Ward of admission (%)						
General medicine	50.8	30.0		54.2	ns	
Long-term care	35.8	30.0		37.5		
Intensive care unit	0.0	10.0	ns	4.2		
Surgery	4.4	10.0		0.0		
Others	9.0	20.0		4.2		
Comorbidity (%)						
Chronic obstructive pulmonary disease	46.3	50.0	ns	37.5	ns	
Heart failure	35.8	50.0	ns	54.2	ns	
Chronic renal insufficiency	22.4	70.0	<0.01	54.2	<0.01	
Diabetes	28.4	40.0	ns	33.3	ns	
Malignant disease	9.0	0.0	ns	25.0	ns	
Inflammatory bowel disease	9.0	10.0	ns	4.2	ns	
Gastrointestinal surgery	13.4	40.0	ns	4.2	ns	
Use of nasogastric tube feeding during the hospital stay	11.9	10.0	ns	4.2	ns	
Previous hospitalization in the 30 days before CDI	28.3	70.0	ns	37.5	ns	
Having at least one of the previous comorbidities	86.6	100.0	ns	91.7	ns	
Global comorbidity index (mean ± SD)	1.8 ± 1.2	2.4 ± 1.1	ns	2.3 ± 1.2	ns	
Medication exposures in the 30 days before CDI (%)						
Any antibiotic not directed at CDI	73.1	90.0	ns	79.2	ns	
Proton pump inhibitors	77.6	80.0	ns	75.0	ns	
Histamine 2 blockers	11.9	20.0	ns	12.5	ns	
**Clinical characteristics and outcomes (%)**						
Leukocyte count ≥ 20 × 10^9^ cells/L *	17.9	10.0	ns	29.2	ns	
Creatinine ≥ 2 mg/dL *	10.8	50.0	<0.01	50.0	<0.01	
Albumin ≤ 2.5 mg/dL *	22.6	12.5	ns	44.4	ns	
Intra-hospital all-cause mortality	4.5	20.0	ns	100.0	<0.01	

ns: not significant; IQR: interquartile range; * within four days before or two days after CDI; **^†^** non-severe and non-recurrent CDI cases are the reference group.

**Table 6:** Association between severe CDI and recurrent CDI and risk factors: results of multinomial logistic regression.

Patients’ Features	Recurrent Cases vs. Non-Severe and Non-Recurrent CDI			Severe Cases vs. Non-Severe and Non-Recurrent CDI		
	OR	95% CI		OR	95% CI	
Age	1.00	0.93–1.08	ns	0.95	0.86–1.04	ns
Creatinine						
≥2 mg/dL	7.15	2.03–25.16	0.002	5.33	1.02–27.93	0.048
<2 mg/dL	reference			reference		
Any antibiotic not directed at CDI **^†^**						
yes	0.62	0.16–2.50	ns	2.14	0.18–25.79	ns
no	reference			reference		
Leukocyte count						
≥20 × 10^9^ cells/L *	2.15	0.60–7.65	ns	0.52	0.05–5.17	ns
<20 × 10^9^ cells/L *	reference			reference		
Histamine 2 blocker **^†^**						
yes	0.65	0.11–3.74	ns	1.04	0.11–10.33	ns
no	reference			reference		
Proton pump inhibitor **^†^**						
yes	0.94	0.23–3.85	ns	1.35	0.18–9.87	ns
no	reference			reference		
Chronic renal insufficiency						
yes	3.07	0.84–11.19	ns	6.20	1.09–35.41	0.040
no	reference			reference		
Use of nasogastric tube feeding during the hospital stay						
yes	0.39	0.03–4.54	ns	0.65	0.32–12.92	ns
no	reference			reference		

ns: not significant; IQR: interquartile range; * within four days before or two days after CDI; **^†^** during the 30 days before CDI.

## Abbreviations

The following abbreviations are used in this manuscript:

CDI	*Clostridium difficile* infections
IQR	Interquartile range
LIS	Laboratory Information System
SIMPIOS	Società Italiana Multidisciplinare Prevenzione Infezioni Organizzazioni Sanitarie
